# Importance of real-time RT-PCR to supplement the laboratory diagnosis in the measles elimination program in China

**DOI:** 10.1371/journal.pone.0208161

**Published:** 2018-11-30

**Authors:** Aili Cui, Naiying Mao, Huiling Wang, Songtao Xu, Zhen Zhu, Yixin Ji, Li Ren, Lingyu Gao, Yan Zhang, Wenbo Xu

**Affiliations:** WHO WPRO Regional Reference Laboratory of Measles/Rubella and Key Laboratory of Medical Virology Ministry of Health, National Institute for Viral Disease Control and Prevention, Beijing, People’s Republic of China; Kliniken der Stadt Köln gGmbH, GERMANY

## Abstract

In addition to high vaccination coverage, timely and accurate laboratory confirmation of measles cases is critical to interrupt measles transmission. To evaluate the role of real-time reverse transcription-polymerase chain reaction (RT-PCR) in the diagnosis of measles cases, 46,363 suspected measles cases with rash and 395 suspected measles cases without rash were analyzed in this study; the cases were obtained from the Chinese measles surveillance system (MSS) during 2014–2017 and simultaneously detected by measles-specific IgM enzyme-linked immunosorbent assay (ELISA) and real-time RT-PCR. However, some IgM-negative measles cases were identified by real-time RT-PCR. The proportion of these IgM-negative and viral nucleic acid-positive measles cases was high among measles cases with measles vaccination history, cases without rash symptoms, and cases within 3 days of specimen collection after onset. The proportion of IgM-negative and viral nucleic acid-positive measles cases in the 0–3 day group was up to 14.4% for measles cases with rash and 40% for measles cases without rash. Moreover, the proportions of IgM-negative and nucleic acid-positive measles cases gradually increased with the increase in the measles vaccination dose. Therefore, integrated with IgM ELISA, real-time RT-PCR would greatly improve the accurate diagnosis of measles cases and avoid missing the measles cases, especially for measles cases during the first few days after onset when the patients were highly contagious and for measles cases with secondary vaccine failure. In conclusion, our study reconfirmed that IgM ELISA is the gold-standard detection assay for measles cases confirmation. However, real-time RT-PCR should be introduced and used to supplement the laboratory diagnosis, especially in the setting of pre-elimination and/or elimination wherever appropriate.

## Introduction

Measles is a highly contagious viral disease that causes enormous morbidity and mortality among children in the prevaccine era. As a vaccine-preventable disease, a measles epidemic could be effectively controlled and interrupted using a safe and effective vaccine. Under the Global Vaccine Action Plan, measles and rubella are targeted for elimination in five WHO Regions by 2020 [1]. To achieve measles elimination, in addition to maintaining high vaccination coverage, a sensitive case-based surveillance system is also considered to be very important. For the low-incidence or elimination phase, case-based surveillance should be conducted, and every case should be reported and investigated immediately. Thus, an accurate diagnosis for every measles case is critical for measles elimination, and laboratory specimens should be collected from every sporadic/outbreak case to characterize viral circulation and importation patterns [2].

China has made substantial efforts to achieve the measles elimination goal in recent years, including the implementation of high vaccination coverage through routine and supplementary immunization activities, as well as by conducting a high-quality epidemiological and laboratory measles surveillance [3]. Through these efforts and measures, the incidence of reported cases of measles has drastically reduced in recent years [3,4]. In 2017, the incidence of measles has reached the lowest level in history (unpublished data). The Chinese Measles Laboratory Network (CMLN) was established in 2001 and is composed of one national, 32 provincial and 339 prefectural laboratories [5]. The measles surveillance system (MSS), as a national case-based system of measles surveillance with laboratory support, has been implemented since 2009 [3].

In China, 53.0% of serum samples were collected within 3 days of rash onset for the laboratory test. Based on the China national measles surveillance guideline, second serum samples were required to be collected if the first serum sample was negative. However, the second serum samples were very difficult to follow up with unconfirmed measles cases who can freely travel anywhere after seeing the doctor for the first time, and very few second serum samples were obtained for laboratory detection. Therefore, real-time reverse transcription-polymerase chain reaction (RT-PCR) has been widely introduced into CMLN to supplement the laboratory test, in addition to IgM ELISA, based on the updated national measles surveillance guideline since 2014. In this study, we focused on the evaluation of the role of real-time RT-PCR in the measles pre-elimination phase, compared with the IgM ELISA test, based on the measles surveillance data obtained from MSS during 2014–2017.

## Materials and methods

### Case definitions

Suspected measles cases in measles surveillance were defined as any person with fever and rash and one or more of the following symptoms: cough, coryza and conjunctivitis, according to the China national measles surveillance guideline. Additionally, the cases suspected as measles cases by the doctor responsible for the report were also included in the measles surveillance system, and these cases may show no rash among their clinical symptoms [3]. Suspected measles cases were confirmed as by laboratory testing, epidemiological linkage or clinical criteria (http://www.wpro.who.int/immunization/documents/measles_elimination_field_guide_2013.pdf?ua=1) [3].

### Dataset

In accordance with the China national measles surveillance guideline of 2014, the investigations of suspected measles cases were carried out, and information was collected by MSS. In this study, case-based measles surveillance data during 2014–2017 were downloaded from MSS, including the number of suspected measles cases, the vaccination status, specimen collection information, and laboratory test results. The suspected measles cases tested simultaneously with measles-specific IgM ELISA and real-time RT-PCR were selected for further analysis. Meanwhile, the cases with incomplete information regarding the vaccination status and specimen collection date were excluded from the dataset. Vaccination status was classified as 0 dose, 1 dose, 2 doses, and ≥3 doses. The number of days of specimen collection after onset was classified according to the four groups: 0–3, 4–5, 6–10, and >10. Case numbers were counted by the date of onset, and the positive rate and proportion of measles-specific IgM ELISA and real-time RT-PCR were calculated using Microsoft Excel.

Additionally, the dataset of suspected measles cases without rash was prepared in the same way. The results of measles-specific IgM ELISA and real-time RT-PCR were analyzed in this study.

### Quality control of the laboratory test

Based on the China national measles surveillance guideline, the prefecture laboratories were responsible to collect the serum specimens and pathogenic specimens (including throat swab or urine) from the suspected measles cases and subject them to measles-specific IgM ELISA and real-time RT-PCR [4]. The pathogenic specimens including throat swabs or urine were collected and stored in a cold chain or -70 freezer during transportation and storage. Additionally, the pathogenic specimens were required to be collected as early as possible after rash onset [6]. The validated commercial kits were used in the laboratory detection for suspected measles cases. The commercial IgM ELISA kits mostly used in the laboratory network in China were the Haitai Measles IgM antibody detection kit (Haitai Biotechnology, Zhuhai, China) and Virion/Serion Measles virus IgM kit (Institute Virion/Serion GmbH, Würzburg, Germany). The commercial real-time RT-PCR kits used in the laboratory network in China included the Shuoshi measles virus real-time RT-PCR kit (BioPerfectus technologies, Jiangsu, China), Jinhao measles virus RNA real-time RT-PCR detection kit (Beijing Kinghawk Pharmaceutical company, Beijing, China) and Zhongshan Daan measles virus RNA real-time RT-PCR detection kit (Zhongshan University Daan Gene Technology Corp, China).

To ensure high-quality laboratory testing in the network laboratories, CMLN implemented a comprehensive quality control system, including hands-on training and technical support, on-site review, and proficiency tests (PTs) for both serology and nucleic acid detection assays annually, including but not limited to ELISA IgM and real-time RT-PCR. Since 2014, molecular proficiency panels were sent to all 32 provincial laboratories to assess their ability to perform real-time RT-PCR, RT-PCR and genotyping once every two years. Each province regularly performed the same quality control measures for their administered prefectural laboratories following the national laboratory guidance.

### Ethics statement

In this study, the only human materials used were sera, throat swabs and urine specimens collected from the clinically suspected measles patients for the purpose of public health and disease control. This study was approved by the second session of the Ethics Review Committee of the National Institute for Viral Disease Control and Prevention in the China CDC, and the methods were performed in accordance with the approved guidelines.

## Results

### Laboratory confirmation for suspected measles cases

In total, 338,644 suspected measles cases were investigated from 2014 to 2017 (Table 1). Serum and pathogenic specimens were collected and tested from 307,056 cases (90.7%) and 98,699 cases (29.1%), respectively. These specimens were tested by IgM ELISA and/or real-time RT-PCR. Additionally, for some suspected measles cases, the serum and pathogenic specimens were simultaneously collected and tested by both methods. 53.0% of sera and 63.3% of pathogenic specimens were collected within 3 days after rash onset for measles-specific IgM ELISA and real-time RT-PCR, respectively. The total positive rates of the IgM test and real-time RT-PCR during 2014–2017 were 36.0% and 29.6%, respectively (Table 1). Except for 2014, the positive rates of real-time RT-PCR were similar to those of measles-specific IgM ELISA test during 2015–2017.

**Table 1:** Summary of the suspected measles cases from MSS during 2014–2017.

Year	No. of suspected cases	Measles IgM test		Real-time RT-PCR test		No. of confirmed measles cases			
		No. of cases	No. of Positive (%)	No. of cases	No. of Positive (%)	Laboratory-confirmed	Epidemiologically confirmed	Clinically confirmed	Total cases
2014	127,554	111,432	45,805 (41.1%)	17,889	5,137 (28.7%)	48,344	136	4,184	52,664
2015	104,948	96,918	37,555 (38.7%)	38,548	13,766 (35.7%)	40,717	161	1,504	42,382
2016	69,288	64,123	22,135 (34.5%)	26,098	8,534 (32.7%)	23,916	44	879	24,839
2017	36,854	34,583	5,067 (14.7%)	16,164	1,755 (10.9%)	5,131	50	812	5,993
Total	338,644	307,056	110,562 (36.0%)	98,699	29,192 (29.6%)	118,108	391	7,379	125,878

During 2014–2017, 125,878 measles-confirmed cases were reported in MSS, including 118,108 for laboratory-confirmed cases, 391 for epidemiologically confirmed cases, and 7,379 for clinically confirmed cases (Table 1).

### Serological and nucleic acid confirmation among suspected measles cases with rash

Excluding the cases with incomplete information of the vaccination status and specimen collection date, 46,363 suspected measles cases with rash detected simultaneously by both methods were selected from 338,644 suspected cases for further analysis. In total, 15,999 of the 46,363 cases were confirmed as measles cases by measles-specific IgM ELISA or real-time RT-PCR, and the total positive rate of laboratory-confirmed measles cases was 34.5%. Additionally, 30,364 cases (65.5%) were discarded measles cases, which were negative for both measles-specific IgM and viral nucleic acid (Table 2). Among the 46,363 suspected measles cases, the double-positive rate for measles-specific IgM and viral nucleic acid was 23.7% (11,011/46,363), the single-positive rate for measles-specific IgM was 6.5% (3,028/46,363), and the single-positive rate for viral nucleic acid was 4.2% (1,960/46,363). The proportion of double positive for measles-specific IgM and viral nucleic acid, single positive for measles-specific IgM, single positive for viral nucleic acid were 68.8% (11,011/15,999), 18.9% (3,028/15,999), and 12.3% (1,960/15,999), respectively.

**Table 2:** Results of measles cases with rash by the measles-specific IgM test and real-time RT-PCR.

		Days after rash onset					Doses of measles vaccination				
		0–3	4–5	6–10	〉10	Total	0 dose	1 dose	2 doses	≥3 doses	Total
No. of suspected measles cases	IgM+,Nc+	7,129	2,887	910	85	11,011	8,968	1,389	581	73	11,011
	IgM+,Nc-	1,661	931	357	79	3,028	1,773	856	348	51	3,028
	IgM-,Nc+	1,479	375	84	22	1,960	1,200	480	232	48	1,960
	IgM-,Nc-	18,881	9,386	1,750	347	30,364	9,339	6,222	13,015	1,788	30,364
	Total	29,150	13,579	3,101	533	46,363	21,280	8,947	14,176	1,960	46,363
Total positive rates*		35.2%	30.9%	43.6%	34.9%		56.1%	30.5%	8.2%	8.8%	

* Total positive rates indicate the positive rates for measles-specific IgM ELISA and/or real-time RT-PCR.

Nc: indicates real-time RT-PCR.

**Fig 1:**
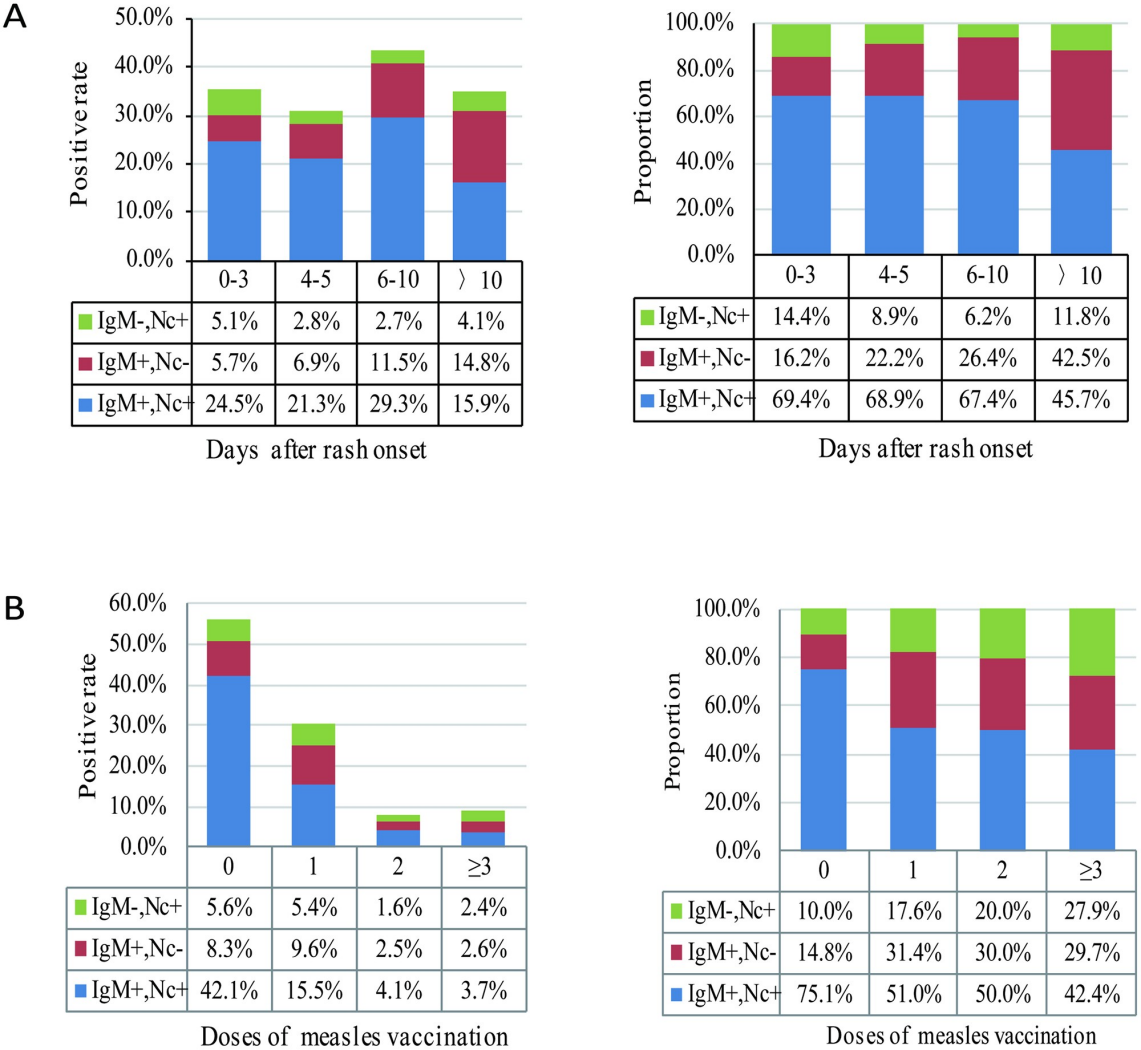
Positive rates and proportion of measles cases with rash by both methods. A: According to the sample collection date after rash onset. B: According to the vaccination status. Nc: indicates real-time RT-PCR.

For the four groups of 0–3, 4–5, 6–10, and >10 days after rash onset, the total positive rates of measles cases ranged from 30.9% to ~43.6% among 46,363 suspected measles cases. Among them, the total positive rate of measles cases in 6–10 days after rash onset was the highest (43.6%) (Table 2). The positive rates of the IgM-negative and nucleic acid-positive measles cases ranged from 2.7% to ~5.1% among 46,363 suspected measles cases, and the proportions were 6.2%~14.4% among 15,999 laboratory-confirmed measles cases in different groups of days after rash onset (Fig 1A). Among them, the positive rate of IgM-negative and nucleic acid-positive measles cases in 0–3 days after rash onset was highest (5.1%), and the proportion in the laboratory-confirmed measles cases was up to 14.4%.

Additionally, 4,058 of 15,999 (25.4%) measles cases have a history of measles vaccination. Regarding the different measles vaccination statuses, the total positive rates of measles cases gradually decreased from 56.1% to 8.2% with the increase in the measles vaccination dose (Table 2). The total positive rate was highest and up to 56.1% in unvaccinated suspected measles cases, and the proportions of IgM-negative and nucleic acid-positive measles cases were also gradually increased from 10.0% to 27.9% with the increase in the measles vaccination dose (Fig 1B). Among 4,058 measles cases with rash and a vaccination history, 760 (18.7%) fell into the group of IgM negative and real-time RT-PCR positive.

**Fig 2:**
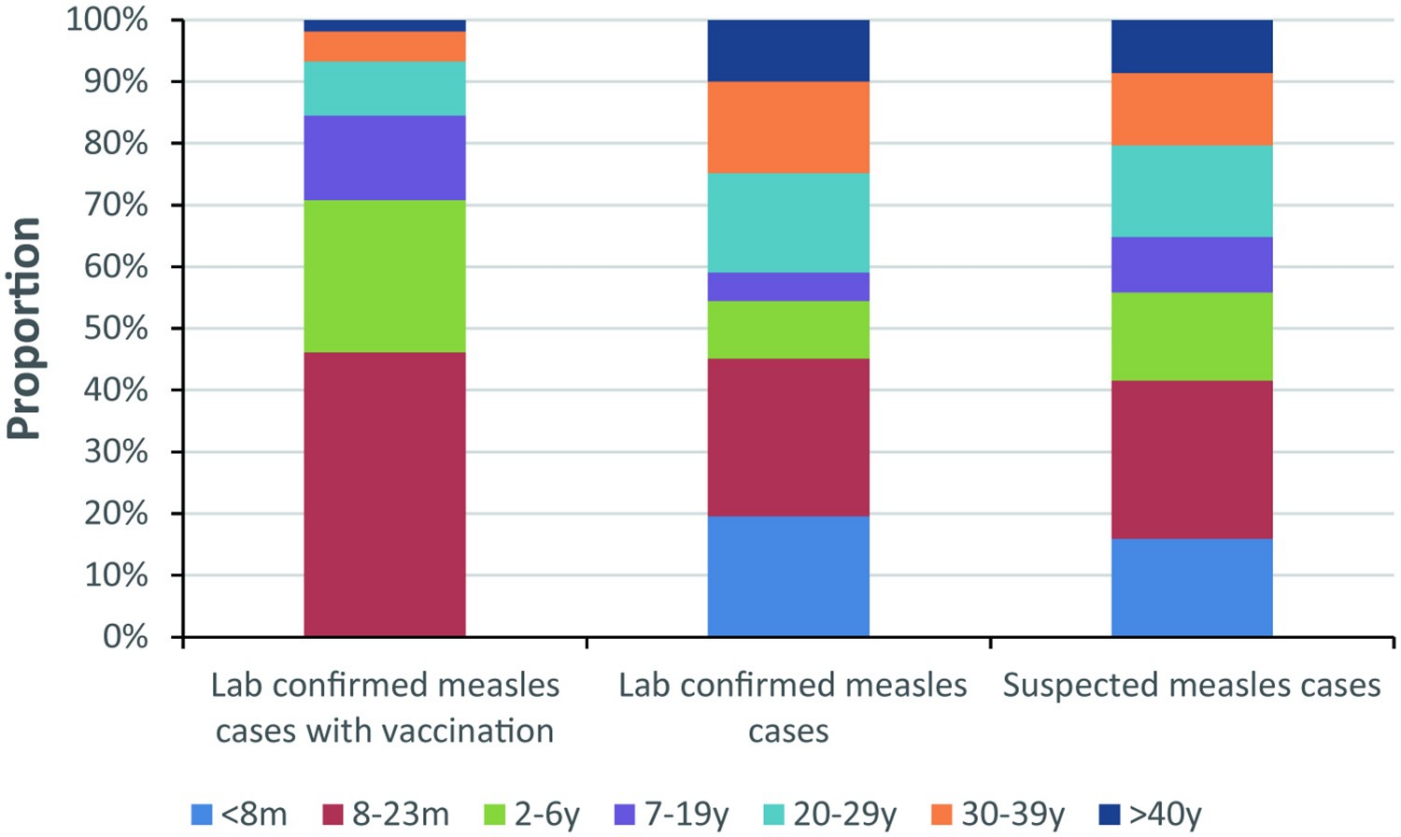
Age distribution among 4,058 laboratory-confirmed measles cases. The age distribution of 4,058 measles cases with a vaccination history was compared with the entire group of suspected and laboratory-confirmed measles cases.

The age distribution of the 4,058 measles cases with a vaccination history mainly comprised on infants and children aged 8–23 months (46.0%), 2–6 years (24.7%) and 7–19 years (13.7%) compared with the entire group of suspected and laboratory-confirmed measles cases (Fig 2). Among the 8- to 23-month group, more than 90% of measles cases had one dose of measles vaccination.

### Serological and nucleic acid confirmation among suspected measles cases without rash

In total, 395 suspected measles cases without rash were collected in this study that were detected simultaneously with measles-specific IgM ELISA and real-time RT-PCR. Among these 395 suspected cases (24.1%), 95 were laboratory-confirmed measles cases, and 300 (75.9%) were discarded measles cases (Table 3). Among the 395 suspected measles cases without rash, the double-positive rate of measles-specific IgM and viral nucleic acid was 9.6% (38/395), the single-positive rate for measles-specific IgM was 7.3% (29/395), and the single-positive rate for viral nucleic acid was 7.1% (28/395). However, among the 95 laboratory-confirmed cases without rash, the proportion of double positive for measles-specific IgM and viral nucleic acid, single positive for measles-specific IgM, and single positive for viral nucleic acid were 40.0% (38/95), 30.5% (29/95), and 29.5% (28/95), respectively. Compared with measles cases with rash, the proportion of positive measles-specific IgM were declined from 87.7% to 70.5%, including double-positive measles cases for measles-specific IgM and viral nucleic acid and single-positive measles cases for measles-specific IgM among the measles cases without rash.

**Table 3:** Results of measles cases without rash by the measles-specific IgM test and real-time RT-PCR.

		Days after rash onset					Doses of measles vaccination				
		0–3	4–5	6–10	〉10	Total	0 dose	1 dose	2 doses	≥3 doses	Total
No. of suspected measles cases	IgM+,Nc+	12	16	8	2	38	26	6	6	0	38
	IgM+,Nc-	14	9	4	2	29	14	6	8	1	29
	IgM-,Nc+	9	7	8	4	28	10	7	7	4	28
	IgM-,Nc-	170	69	45	16	300	97	63	126	14	300
	Total	205	101	65	24	395	147	82	147	19	395
Total positive rates*		17.1%	31.7%	30.8%	33.3%		34.0%	23.2%	14.3%	26.3%	

* Total positive rates indicate the positive rates for measles-specific IgM ELISA and/or real-time RT-PCR.

Nc: indicates real-time RT-PCR.

**Fig 3:**
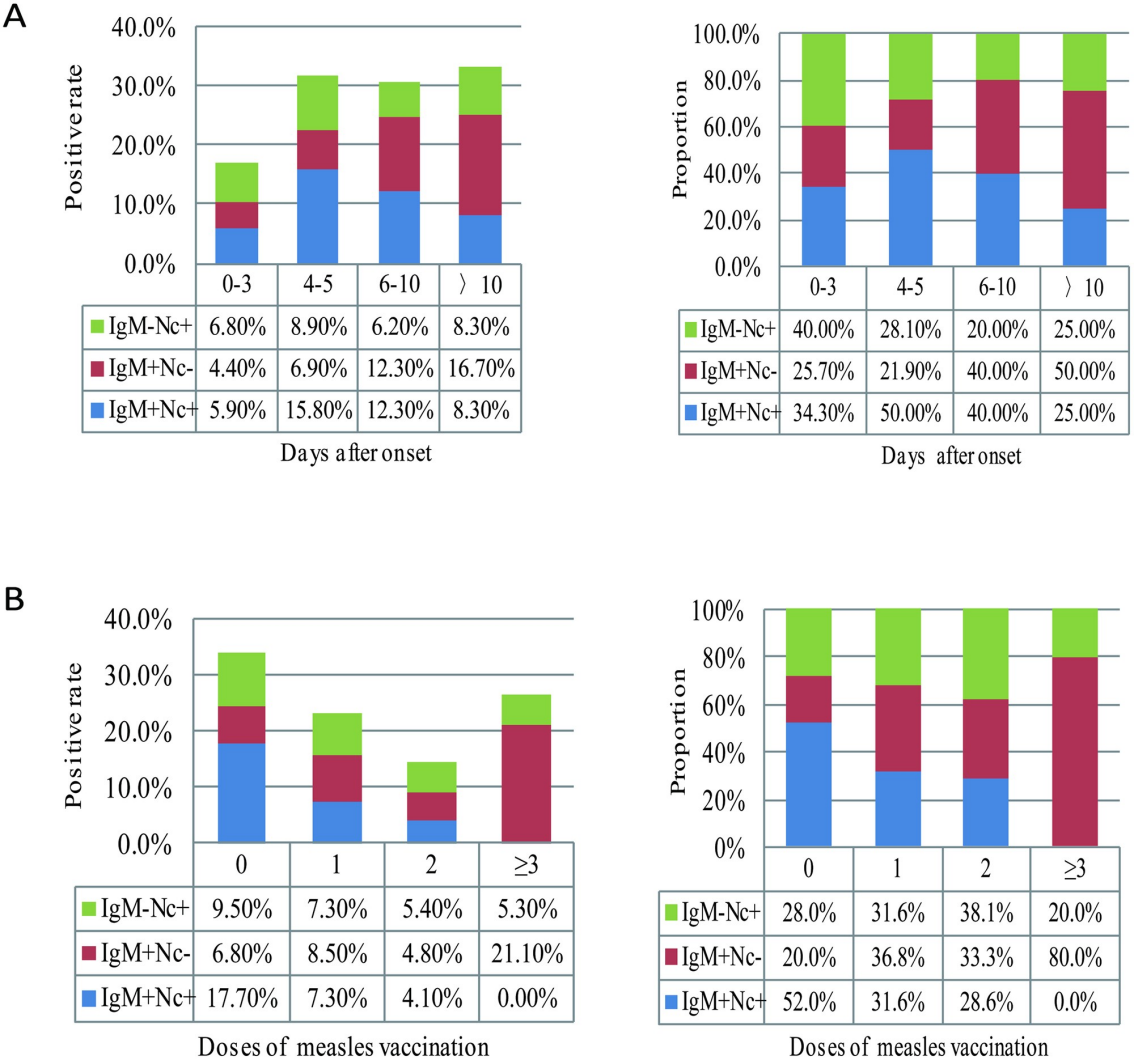
Positive rates and proportion of measles cases without rash by both methods. A: According to the sample collection date after onset. B: According to the vaccination status. Nc: indicates real-time RT-PCR.

For four groups of 0–3, 4–5, 6–10, and >10 days after onset, the total positive rates ranged from 17.1% to ~33.3% among the suspected measles cases without rash (Table 3). Moreover, the total positive rate in 0–3 days after onset was lower than the others at only 17.1%. The proportions of IgM-negative and nucleic acid-positive measles cases were 20.0%~40.0%, which were higher than those of measles cases with rash (6.2%~14.4%) (Fig 3A). Moreover, the proportion of the IgM-negative and nucleic acid-positive measles cases was up to 40.0% among laboratory-confirmed measles cases in 0–3 days after onset.

Additionally, 45 of 95 (47.4%) confirmed measles cases had a history of measles vaccination. Regarding different measles vaccination statuses, the total positive rates ranged from 14.3% to 34.0% (Table 3). The total positive rate was the highest and up to 34.0% in unvaccinated measles cases. The proportions of the IgM-negative and nucleic acid-positive measles cases gradually increased from 28.0% to 38.1% with the increase in the measles vaccination dose (Fig 3B). Due to the limited number, the detection results of ≥3 doses measles cases without rash were not analyzed in this study. Among 45 measles cases without rash and a vaccination history, 18 measles cases (40.0%) fell into the group of IgM negative and real-time RT-PCR positive.

## Discussion

Currently, real-time RT-PCR has widely been used in measles diagnosis for the detection of viral RNA in clinical specimens [7–10]. In measles surveillance, ELISA for measles-specific IgM is considered a gold standard for the confirmation of measles cases [11]. However, it was found that IgM ELISA is more sensitive between days 4 and 28 after rash onset [2]. Up to 30% of measles sera within the first 3 days after rash onset were detected as negative for measles-specific IgM, possibly leading to false-negative results [12]. Additionally, some vaccinated individuals might be infected by the measles virus because of the waning immunity of measles-protective antibodies [13]. These measles cases might show no rash and no typical symptoms, easily leading to a clinical misdiagnosis. In this study, it was found that the proportion (47.4%) of measles vaccination history among measles cases without rash was higher than that of measles cases with rash (25.4%). Meanwhile, measles-specific IgM titers in measles cases with secondary vaccine failure were low or absent, so that IgM antibodies were difficult to be detected by indirect ELISA [14]. To avoid missing measles cases with false-negative IgM detection, real-time RT-PCR is strongly recommended to be implemented in measles laboratory surveillance as a supplementary technique for measles virus confirmation [7].

In this study, the detection results by measles-specific IgM ELISA and real-time RT-PCR among rash cases and no-rash measles cases were analyzed based on the measles surveillance data in China during 2014–2017. Thus, 68.8% of measles cases with rash and 40.0% of measles cases without rash were double positive for measles-specific IgM ELISA and real-time RT-PCR. However, there were a certain number of IgM-negative and viral nucleic acid-positive measles cases among the laboratory-confirmed measles cases. The proportion (29.5%) of IgM-negative and viral nucleic acid-positive measles cases among mild measles cases without rash was higher than that among measles cases with rash (12.3%). The proportions of these IgM-negative and viral nucleic acid-positive measles cases were various in different groups of days after onset and different vaccination statuses. Among them, the proportion in 0–3 days after onset was highest and up to 14.4% for rash cases and 40.0% for no rash cases. Moreover, the proportions of IgM-negative and nucleic acid-positive measles cases gradually increased with the increase in the measles vaccination doses.

If only measles-specific IgM ELISA was applied, many IgM-negative and nucleic acid-positive measles cases would be missed in measles surveillance. Considering the high contagiosity of measles viruses, these missed measles cases would cause measles virus spread and huge challenges for measles elimination. Therefore, viral nucleic acid detection with real-time RT-PCR is very important for measles case confirmation in the measles pre-elimination phase, particularly for measles cases with a history of measles vaccination, no rash symptoms, and a 0- to 3-day collection date after onset. Therefore, integrated with IgM ELISA, real-time RT-PCR would greatly improve the accurate diagnosis of measles cases and avoid missing measles cases, particularly in the measles pre-elimination and elimination stages, when the positive perspective value of confirmed cases is low. Moreover, the early diagnosis of real-time RT-PCR for the cases/specimens collected within the 0- to 3-day collection date after onset would be helpful to interrupt measles virus transmission timely and rapidly control the measles epidemic. Additionally, real-time RT-PCR can be used to screen the positive pathogenic specimen for further genotyping and molecular epidemiology surveillance to support the verification process of elimination. The genotype information of circulating measles viruses is one of three essential criteria for verifying the progress, achievement and maintenance of measles elimination [15]. In countries such as Japan, the USA, Canada and the Republic of Korea, sufficient genotyping information provides evidence to verify the interruption of endemic virus transmission [16–19].

To be line with the WHO measles elimination goal in the world, for countries where endemic measles viruses still circulate, the important genetic baseline data should be established continuously and extensively, to cover continuous years and representative geographic areas as much as possible. Thus, real-time RT-PCR would be very useful to screen the positive pathogenic specimens for further genotyping.

In China, 53.0% of serum samples were collected within 3 days of rash onset for laboratory tests. For these early collection samples, if only the IgM test was performed for case diagnosis, a certain proportion of measles cases would be missed. Moreover, for secondary vaccine failure, cases with waning antibody immunity, the proportion of missed cases would be higher. Worldwide, with the continuous implementation of measles vaccination to support the measles elimination goal, more mild measles cases with secondary vaccine failure might be found, and real-time RT-PCR would be helpful to confirm these measles cases.

China is currently in the measles pre-elimination phase. Although China has made substantial efforts toward measles elimination in recent years, endemic measles cases still circulate in many provinces. As a predominant genotype, the H1 genotype was continually prevalent in China for over twenty years [20,21]. Additionally, a very small number of imported measles virus strains were found using a sensitive virologic surveillance system. Genotypes B3, D4, D8, D9, D11, G3 and H2 were detected since virologic surveillance initiated in 1993 [4,20,22–26]. Timely detection for these imported measles cases were essential for controlling outbreaks and interrupting the virus transmissions in China. In addition to serving as a supplement to case confirmation, real-time RT-PCR could also serve as an effective and practical screening approach to identify the positive pathogenic samples for further virologic surveillance. Continuous and comprehensive virologic surveillance data are one of three essential criteria to verify the process of measles elimination (http://www.wpro.who.int/immunization/documents/measles_elimination_verification_guidelines_2013/en/).

With high vaccination coverage, most measles transmissions have been effectively interrupted in China. Additionally, the overall diversity of measles virus sequences from China has decreased in recent years, coincident with a substantial decrease in measles cases [5,22]. However, measles cases still occur, even among the immunized individuals due to the waning immunity of the measles antibody. As showed in this study, 18.7% of measles cases had rash and 40.0% of measles cases had no rash, but had a vaccination history, falling into the group of IgM-negative and real-time RT-PCR positive. It is very necessary to introduce real-time RT-PCR to the measles surveillance system in the pre-elimination and elimination setting to increase the timely and accurate diagnosis for suspected measles cases that is critical to identify and confirm measles cases in the early phase of outbreaks or sporadic cases and interrupt measles spread.

In this study, measles cases with a vaccination history were found in the age group of 8- to 23-month infants at a proportion of 46.0%, and more than 90% of 8- to 23-month measles cases had one dose of measles vaccination. This finding indicated that the delay of the second dose of routine vaccination is associated with the infection of measles viruses. Regarding the current vaccination schedule in China, 8 months is for the first dose and 18 months for the second dose. However, most of the adult cases were not included in this age distribution analysis because an unknown vaccination history was reported for most of these cases. Thus, a bias of the age distribution of measles cases with vaccination history might be induced in this study.

Furthermore, many discarded measles cases were detected in measles surveillance during 2014–2017. It is well known that other viruses also cause fever and rash illnesses, such as human parvovirus, rubella, EBV or human herpesvirus 6 infections [27–29]. The false-positive IgM results might occur because of nonspecific reaction, interference of rheumatoid factors, or other viruses [11,30]. In an elimination setting, where the positive predictive value of the ELISA is lower than that in endemic and outbreak situations, false-positive results become an important issue necessitating further laboratory testing. Thus, real-time PCR could be used as a supplementary method to confirm the IgM false-positive cases in an elimination setting.

To screen measles cases most effectively, the detection of real-time RT-PCR was implemented in CMLN since 2014. The strong quality control system in China ensures the high quality of testing in each laboratory of the entire laboratory network [4]. Through the four-year implementation of real-time RT-PCR, it was proven that it was effective and reliable for measles case confirmation to support measles surveillance and elimination programs.

In conclusion, our study reconfirmed the IgM ELISA as the gold-standard detection assay for measles case confirmation. However, real-time RT-PCR should be introduced and used as a supplementary laboratory diagnosis in other countries/regions, especially in the setting of pre-elimination and/or elimination wherever appropriate, although the collection and transportation of high-quality pathogenic specimens are huge challenges in some regions/countries in the world. The financial support and technical trainings should be allocated and arranged to implement molecular testing and ensure the accuracy of the testing through a high-quality control system.
